# Definitive Radiotherapy for Patients With Anal Squamous Cell Carcinoma: A Retrospective Cohort Study

**DOI:** 10.7759/cureus.18732

**Published:** 2021-10-13

**Authors:** Atsuto Katano, Hideomi Yamashita

**Affiliations:** 1 Radiology, The University of Tokyo Hospital, Tokyo, JPN

**Keywords:** squamous cell carcinoma, definitive treatment, chemotherapy, radiotherapy, anal carcinoma

## Abstract

Background

Anal squamous cell carcinoma accounts for less than 2-3% of all digestive system carcinomas. The present study aimed to determine the clinical characteristics, treatment patterns, and treatment outcomes of patients at our institution.

Methodology

We reviewed the clinical data of all consecutive patients with anal squamous cell carcinoma who were treated with definitive radiotherapy in our department between July 2009 and July 2020. Radiotherapy was delivered in 1.8-2 Gy daily fractions to a whole pelvic dose ranging from 45 to 50 Gy, followed by boost radiotherapy of 10-15 Gy, resulting in a total dose of approximately 60 Gy. Concurrent chemotherapy with radiotherapy included 5-fluorouracil/mitomycin C or 5-fluorouracil/cisplatin.

Results

A total of 14 patients with a median age of 61.5 years (range: 45-85 years) were analyzed. There were nine women and five men. The clinical T stage was T1 in two patients, T2 in six patients, T3 in two patients, and T4 in four patients. The clinical N stage was N0 in four patients and N1 in 10 patients. Patients with clinical stage III disease comprised 79% of the entire study population. For the entire cohort, the five-year overall survival rate was 83.3% and the five-year progression-free survival rate was 48.5%. One patient experienced grade 3 fecal incontinence, and the others experienced no radiation-induced severe delayed adverse events.

Conclusions

The results of our study demonstrated that definitive radiotherapy with or without chemotherapy for patients with anal squamous cell carcinoma is an effective and feasible treatment.

## Introduction

Anal carcinoma, a rare disease, accounts for only 2.6% of all digestive system carcinomas. The incidence of anal carcinoma among women is approximately twice that of men [1]. Its incidence is gradually increasing in both men and women [2,3]. Chronic human papillomavirus (HPV) infection is the most significant risk factor for anal carcinoma [4]. Oncogenic HPV subtypes have been identified as HPV 16, 18, 31, and 33. Among these, HPV 16 is the most frequent type and is considered a crucial factor for the development of squamous cell carcinoma [5]. Anal intraepithelial neoplasia and anal squamous intraepithelial lesions are considered premalignant signs of anal squamous cell carcinoma, which is driven by HPV infection [6]. Another risk factor is immunosuppression caused by acquired immune deficiency syndrome caused by human immunodeficiency virus (HIV) infection [7]. The incidence of anal cancer is significantly higher in the HIV-positive population than in the general population [8]. Other risk factors include smoking, multiple sexual partners, and a history of gynecological cancer [9-11].

Signs and symptoms of anal cancer often do not occur until the advanced stage. These include anal bleeding, nodule, pain, and changes in bowel habits [12]. These symptoms are similar to those of other benign diseases such as hemorrhoids and anorectal fistulae. Some patients present with locally advanced tumors at the time of diagnosis. Tanum et al. reported that one-third of the patients experienced more than six months delay in diagnosis [13].

Concurrent chemoradiotherapy is considered the standard treatment for anal squamous cell carcinoma. However, few studies from Japan have reported the clinical outcomes of patients with anal cancer treated with definitive radiotherapy. Takashima et al. reported a low proportion of patients with anal squamous cell carcinoma who underwent chemoradiotherapy in Japan [14]. The present study aimed to determine the clinical characteristics, treatment patterns, and treatment outcomes of patients at The University of Tokyo Hospital.

## Materials and methods

We reviewed the clinical data of all consecutive patients with anal squamous cell carcinoma treated with definitive radiotherapy in our department between July 2009 and July 2020. The treatment modalities included in the study were radiotherapy alone and concurrent chemoradiotherapy for definitive intent. We excluded the following cases: the presence of distant metastases, a history of radiotherapy for anal carcinoma before current treatment, and no intention to undergo definitive radiotherapies, such as palliative or neoadjuvant intention. The data for all patients were obtained and reclassified based on the criteria of the American Joint Committee on Cancer/Union for International Cancer Control, tumor, node, metastasis staging manual (eighth edition). Adverse events were classified by grade according to the Common Terminology Criteria for Adverse Events version 5.0. The study was conducted in accordance with the Declaration of Helsinki and was approved by the Research Ethics Committee of the Graduate School of Medicine and Faculty of Medicine of the University of Tokyo (study protocol number: 3372-6). All patients provided written informed consent.

All patients were treated with three-dimensional conformal radiation therapy (3D-CRT) or intensity-modulated radiation therapy (IMRT). Furthermore, all patients underwent a planning computed tomography (CT) scan in the treatment position; radiotherapy planning was based on a 5-mm or 2-mm CT slice thickness for 3D-CRT or IMRT, respectively. Gross tumor volume (GTV) was defined as visible primary tumor and positive metastatic lymph node in treatment planning CT, pretreatment magnetic resonance imaging (MRI), or positron emission tomography scan. Clinical target volume (CTV) was defined as GTV with a clinical margin combined with inguinal, external iliac, internal iliac, presacral, and mesorectal lymph nodes. The planning target volume (PTV) was defined as GTV and CTV with a 3-5 mm uniform expansion to account for the daily setup margin. Radiotherapy was delivered in 1.8-2 Gy daily fractions to a whole pelvic dose ranging from 45 to 50 Gy, followed by boost radiotherapy of 10-15 Gy, resulting in a total dose of approximately 60 Gy. Radiotherapy planning was performed using the Pinnacle treatment planning system (Philips Medical Systems, Amsterdam, the Netherlands).

For statistical analyses, the open-source software package R (The R Foundation for Statistical Computing, Vienna, Austria) was used. The survival rate was calculated using the Kaplan-Meier method. Overall survival (OS) was defined as the time from diagnosis to the initiation of radiotherapy to death from any cause. Progression-free survival (PFS) was defined as the time from the initiation of radiotherapy to any disease progression or death from any cause. Locoregional control (LRC) was defined as the time from the initiation of radiotherapy to freedom from locoregional progression. Statistical significance was set at p < 0.05.

## Results

A total of 14 patients with a median age of 61.5 years (range: 45-85 years) and a median Karnofsky performance status score of 80 (range: 70-90) were retrospectively analyzed in this study. There were more female (n = 9) patients than male (n = 5). Patients with stage III disease (n = 11, 79%) and concurrent administration of 5-fluorouracil (5-FU)/mitomycin C (MMC) (n = 10; 71%) were predominant. The median follow-up duration was 32 months (range: 1-130 months). The 5-FU/MMC regimen typically consisted of two cycles of 5-FU (1,000 mg/m^2^/day on days one to four), along with MMC (10 mg/m^2^ on day one). The 5-FU/cisplatin (CDDP) regimen consisted of two cycles of 5-FU (1,000 mg/m^2^/day on days one to four), along with CDDP (75 mg/m^2^ on day 1).

Radiotherapy was delivered through 3D-CRT (n = 8; 57%) or IMRT (n = 6; 43%). The median dose to whole pelvic radiotherapy was 45 Gy (range: 44-50.4 Gy), and the median dose to boost radiotherapy was 13.5 Gy (range: 9-16 Gy) delivered in 1.8-2 Gy per fraction. The median total radiotherapy dose was 59.4 Gy (range: 54-61 Gy).

**Table 1:** Patient characteristics Patient characteristics of 14 patients treated by radiotherapy for anal squamous cell carcinoma. 5-FU: 5-fluorouracil; MMC: mitomycin C; CDDP: cisplatin; 3D-CRT: three-dimensional conformal radiation therapy; IMRT: intensity-modulated radiation therapy

Variables		Number (Percentage)
Age: Median (Range)		61.5 (range: 45–85)
Gender		
	Male	5 (36%)
	Female	9 (64%)
Karnofsy performance scale		
	90	6 (43%)
	80	7 (50%)
	70	1 (7%)
T stage		
	T1	2 (14%)
	T2	6 (43%)
	T3	2 (14%)
	T4	4 (29%)
N stage		
	N0	4 (29%)
	N1	10 (71%)
Clinical stage		
	IIA	3 (21%)
	IIIA	5 (36%)
	IIIB	1 (7%)
	IIIC	5 (36%)
Concurrent chemotherapy		
	5-FU/CDDP	2 (14%)
	5-FU/MMC	10 (71%)
	None	2 (14%)
Radiation treatment modality		
	3D-CRT	8 (57%)
	IMRT	6 (43%)
Actual delivered number of chemotherapy courses		
	0	2 (14%)
	1	1 (7%)
	2	10 (71%)
	4	1 (7%)
Prescription dose to whole pelvis: Median (Range)		45 Gy (44–50.4 Gy)
Prescription dose to boost: Median (Range)		13.5 Gy (9–16 Gy)
Total prescription dose (Range)		59.4 Gy (54–61 Gy)

**Figure 1:**
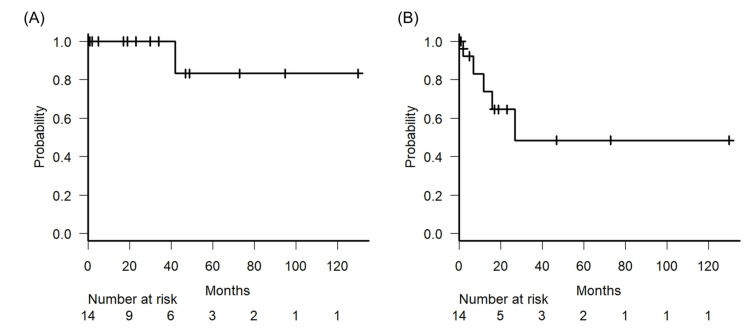
Kaplan-Meier survival curve. (A) Kaplan-Meier survival curve for overall survival rates of 14 patients in our study. (B) Kaplan-Meier survival curve for progression-free survival rates of 14 patients in our study. The vertical bar corresponds to the censored case.

For the entire cohort, the five-year OS was 83.3% (95% confidence interval [CI]: 27.3-97.5%; Figure 1A) and PFS was 48.5% (95% CI: 14.4-76.3%; Figure 1B). Five patients experienced recurrence during the observational period. Among them, four had local recurrences, resulting in a five-year LRC of 58.2% (95% CI: 21.7%). All four patients underwent salvage surgery and underwent permanent colostomy. One patient had distant metastasis recurrence in the mediastinal and abdominal lymph nodes. The patient received no further treatment and chose the best supportive care.

Regarding radiation-induced adverse events, the most frequent acute adverse event was dermatitis (grade 3, n = 3; grade 2, n = 5; grade 1, n= 4), followed by diarrhea (grade 2, n = 6; grade 1, n = 3), anal pain (grade 3, n = 1; grade 2, n = 1), and cystitis (grade 2: n = 1, grade 1: n = 1). Except for one patient, severe radiation-induced late adverse events were not observed. The patient experienced grade 3 fecal incontinence and underwent hyperbaric oxygen therapy.

## Discussion

Anal carcinoma was managed surgically with permanent colostomy until the 1970s. Nigro et al. reported the high efficacy of neoadjuvant chemoradiotherapy before surgery for anal squamous cell carcinoma in 1983 [15]. The average age at diagnosis for anal carcinoma was the early 60s [16]. Their neoadjuvant chemoradiotherapy consisted of intravenous administration of MMC and 5-FU infusion combined with radiotherapy of 15 fractions of 2 Gy to a total dose of 30 Gy. In 1985, Leichman et al. also achieved a high rate of pathological complete response after neoadjuvant chemoradiotherapy [17]. Thus, chemoradiotherapy could avoid colostomies while maintaining good survival rates. Moreover, some phase III trials reported that chemoradiotherapy is therapeutically superior to radiotherapy alone in the treatment of anal carcinoma. The European Organization for Research and Treatment of Cancer Radiotherapy and Gastrointestinal Cooperative Groups reported that chemoradiotherapy was superior in locoregional control and colostomy-free interval compared to radiotherapy alone [18]. The United Kingdom Coordinating Committee on Cancer Research group reported that the chemoradiotherapy group achieved a 46% reduction in the risk of local failure compared to the radiotherapy alone group; moreover, chemoradiotherapy reduced the risk of death from anal cancer (hazard ratio: 0.71, 95% CI: 0.53-0.95; p = 0.02) in the Anal Cancer Trial (ACT) I trial [19].

The standard chemotherapy regimen with radiotherapy for anal cancers is the concurrent administration of MMC and 5-FU. The intergroup Radiation Therapy Oncology Group (RTOG) 8704 trial showed that the addition of MMC to 5-FU combination radiotherapy resulted in a significantly higher disease-free survival rate (73% vs. 51%; p < 0.001) and colostomy-free survival rate (71% vs. 59%; p = 0.014) than 5-FU combination radiotherapy [20]. As a serious clinical problem in this trial, 18% of the patients who were randomized to the 5-FU plus MMC arm experienced grade 4 or 5 hematologic toxicity. The RTOG 9811 trial aimed to evaluate the treatment outcome of replacing MMC with CDDP, expecting enhancement of radiosensitivity by CDDP usage and improvement of hematologic adverse events. However, 5-FU/CDDP did not improve disease-free survival compared to 5-FU/MMC treatment, and the rate of colostomy was significantly higher in the 5-FU/CDDP treatment arm [21,22]. This result was observed because only the 5-FU/CDDP group received two courses of induction chemotherapy. The ACT II trial employed a 2 × 2 factorial design to compare 5-FU+CDDP versus 5-FU/MMC with or without two courses of maintenance chemotherapy [23]. The trial revealed no significant difference between each regimen in terms of clinical outcome and no need for adjuvant treatment. In the ACCORD 03 trial, there was no advantage for either induction chemotherapy or a high-dose radiation boost of 20-25 Gy for locally advanced anal canal carcinoma [24]. In addition, a systematic review concluded that no clinical benefit was proven with the addition of induction or adjuvant chemotherapy [25].

According to the results of the RTOG 9811 trial, five-year PFS, OS, and loco-regional failure rates in patients treated with radiotherapy with concurrent 5-FU+MMC administration were 67.8%, 78.3%, and 20%, respectively. Although PFS and LRC in our study appeared to be inferior to the results, it was because our study included a relatively high proportion of advanced cases compared to the RTOG 9811 trial (locally advanced cases (T3 or T4): 43% vs. 27%, clinically positive lymph nodes: 71% vs. 26%). Moreover, two patients who could not receive concurrent chemotherapy might have a deleterious effect on our results. One of the two patients experienced local relapse approximately two years after chemoradiotherapy.

Although chemoradiotherapy is an effective initial treatment for anal cancer, local progression is the predominant type of recurrence [26]. If biopsy proves local progression, salvage surgery has the potential to achieve good long-term outcomes. Schiller et al. revealed that the five-year OS of 40 highly selected patients with anal cancer who underwent salvage surgery with a permanent colostomy after chemoradiotherapy was 39% [27]. However, salvage surgery for recurrent anal cancer was associated with a high risk for postoperative complications due to the late adverse effects of chemoradiotherapy on wound healing. Accurate patient selection is an essential issue in salvage therapy. In the present study, all patients with local recurrence underwent salvage surgery without severe complications. For patients who cannot tolerate surgery, re-irradiation might be considered as a treatment option. Osborne et al. reported that hyperfractionated accelerated re-irradiation therapy after definitive chemoradiotherapy for anal carcinoma was tolerated without severe late adverse events, resulting in a three-year OS of 60% [28].

According to recent reports from Japan, Tachibana et al. reported excellent clinical outcomes of chemoradiotherapy for anal canal cancer in 13 patients [29]. Their study included two patients with clinical T3/T4 stage, and they reported that the five-year LRC and five-year OS rates were 92% and 100%, respectively. Sakanaka et al. analyzed data from 10 patients with anal cancer, including six patients with clinical stage II who had been treated with simultaneous integrated boost IMRT, reporting three-year PFS and OS of 80% and 100%, respectively [30]. Recently, for better treatment outcomes, durvalumab was added to standard chemoradiotherapy for locally advanced anal carcinoma in a randomized multicenter phase II trial [31]. Jones et al. reported that multiparametric MRI was effective in predicting recurrence in the treatment of squamous cell carcinoma of the anal canal [32].

A major limitation of this study was the insufficient number of cases to reach a statistically significant conclusion. In addition, the present study was a retrospective study with various unavoidable biases. For example, a relatively small number of radiotherapy alone cases was included in the present study.

## Conclusions

The results of our study demonstrated that definitive radiotherapy with or without chemotherapy for patients with anal squamous cell carcinoma is an effective and feasible treatment. These findings are comparable to those previously reported. However, further studies are required to establish better treatment strategies for anal cancer.
